# Exploring the Dynamic Changes of Intercellular Connections in Cervical Cancer: Insights From Transcriptomic Data Combined With Single‐Cell Sequencing

**DOI:** 10.1155/humu/8140041

**Published:** 2026-01-22

**Authors:** Ran Ji, Rui Geng, Zhaoyue Zhang, Feng Gao, Pengpeng Zhang, Ying Sun, Jinhui Liu

**Affiliations:** ^1^ The First Clinical Medical College, Nanjing Medical University, Nanjing, Jiangsu, China, njmu.edu.cn; ^2^ Suzhou Center for Disease Control and Prevention, Suzhou, China; ^3^ Department of Oncology, The Affiliated Suqian First People′s Hospital of Nanjing Medical University, Suqian, Jiangsu, China; ^4^ Department of Radiation Oncology, The First Affiliated Hospital of Nanjing Medical University, Nanjing, China, njmu.edu.cn; ^5^ Department of Osteology, First Affiliated Hospital of Nanjing Medical University, Nanjing, Jiangsu, China, njmu.edu.cn; ^6^ Department of Lung Cancer, Tianjin Lung Cancer Center, National Clinical Research Center for Cancer, Key Laboratory of Cancer Prevention and Therapy, Tianjin′s Clinical Research Center for Cancer, Tianjin Medical University Cancer Institute and Hospital, Tianjin, China, tmucih.com; ^7^ Department of Gynecology, The First Affiliated Hospital of Nanjing Medical University, Nanjing, China, njmu.edu.cn

**Keywords:** cervical carcinoma, immunotherapy, prognosis, tumor microenvironment

## Abstract

**Background:**

As a common gynecological malignancy, cervical cancer has a rising incidence rate and mortality, which has brought huge pressure to global public health. Although immunotherapy has been applied in clinical practice, its therapeutic effect is still far from satisfactory.

**Methods:**

InferCNV was used to calculate the CNV score and the ssGSE, which is an algorithm to calculate the abundance of samples. CellChat analysis and pseudotime analysis were used to observe the evolution and interaction relationships between different clusters. Establish a prognostic model for CC patients using univariate, LASSO, and Cox analysis, and evaluate copy number variation and TME in low‐risk groups. Finally, ssGSEA was applied to calculate the relationship between the hallmark gene sets and immune cycle steps and to calculate drug sensitivity in different risk groups using “oncopredict.” A series of experiments including CCK‐8 assay, clone formation, EdU assay, and Transwell assay were performed to detect the role of COL4A1 in CC.

**Results:**

The epithelial cells were divided into nine clusters. Among them, Cluster 8 has a lower CNV score, a lower degree of variation, and a better prognosis. After that, Cluster 8 sends a signal to fibroblasts through the PTN signaling pathway. A cervical cancer–related model (CCM) was constructed based on the marker genes of Cluster 8, and it can effectively distinguish the prognosis. There is a great difference in standardized TMB, immune cell infiltration, and ESTIMATE scores between the groups. Nine drugs were identified which may achieve better therapeutic effects when applied to low‐risk patients. Finally, knockdown of COL4A1 inhibits the proliferation and metastatic ability of CC cells.

**Conclusion:**

Our study revealed different interactions between subgroups in the tumor microenvironment of CC epithelial cells. We established an effective prognostic model. Ultimately, through a series of in vitro function experiments, COL4A1 was recognized as a new potential target for the therapeutic intervention of CC.

## 1. Introduction

Cervical cancer (CC) is one of the most common gynecological cancers around the world [1]. Through hrHPV screening and Papanicolaou smear testing, the incidence rate of CC in developed countries has declined, while in developing countries, the rate is still rising [2]. Besides its impact on individual health, CC also takes a huge social and economic burden on countries. Main risk factors for CC include hrHPV, age, smoking, oral contraceptives, and diet [3]. Most patients are already in an advanced state at the time of diagnosis and have missed the best surgical time. Despite improvements in the detection and treatment of CC, the prognosis for patients with metastatic and recurrent diseases remains poor. In addition, tumor heterogeneity may lead to resistance to therapy in some CC patients. Therefore, catching a new therapy to improve the prognosis of CC patients is crucial.

CC originates from normal cervical epithelium. The cervix has unique immunological characteristics and undergoes specific changes under different physiological conditions, which can generate immune responses crucial for preventing infection and other harmful environmental damage [4]. More and more evidence proved that lots of cells in cervical tissue are associated with the immune response, among which cervical epithelial cells are the direct counterparts of pathogens [5]. Epithelial cells have a great effect on initiating, maintaining, and regulating innate and adaptive immune function in coordination with immune cells of various tissues, including the cervix. It may prevent progression and kill microorganisms through various means [6]. Essentially, cervical epithelial cells have multiple immune abilities that prevent pathogens from female reproductive tract. In addition, the hrHPV virus causes changes in the microenvironment surrounding squamous intraepithelial lesions, which also plays a driving role in tumor progression [5].

More and more evidence suggests that cancer is closely related to immune microenvironment modifications [4]. Immune checkpoint inhibitors are considered an effective treatment, which has been considered first‐ and second‐line treatment of various cancers by the FDA [7], and have shown good efficacy. Immunotherapy is becoming increasingly important as a treatment for CC, and its effectiveness can be maximized by precisely selecting immune targets [8]. Evaluating the tumor immune microenvironment will be conducive to tumor immunotherapy and finding people who benefit most.

Tumors are complex systems that consist of various cell types. ScRNA‐seq has become a useful tool for revealing the heterogeneity of various cancers by displaying different cell types and cell states in tumors [9, 10]. Due to the different ratios of different cell types in TME, there are differences in the relationship between molecular subtypes and disease prognosis, indicating that tumor heterogeneity is crucial for disease progression [11]. Therefore, a thorough grasp of the heterogeneity and tumoral crosstalk will help identify more effective targets for CC therapy [12]. Despite extensive studies of immune checkpoint inhibitors, the heterogeneity of cell types of CC remains unclear [13]; further research is needed to be conducted.

In this study, we revealed dynamic changes in intercellular connections and various cell subtypes, finding new insight about the molecular basis of CC epithelial cell development. By classifying different cell clusters, we identified their effect on TME. Finally, we built a model by marker genes that can reflect the prognosis of CC patients and their effect on immunotherapy and chemotherapy. This offers new insight into the treatment of CC.

## 2. Materials and Methods

### 2.1. Data Source

Three scRNA‐seq datasets from different sources were used in our research. Six samples (three CC and three adjacent normal tissues) were obtained from E‐MTAB‐11948. RNA sequencing data of 76,911 single cells from 13 human cervical tissue samples at different disease stages were obtained from a published paper, including 10 samples with cervical abnormalities (two with CIN, three with CESC, and five with advanced CESC) and 3 NC from the ectocervix [14]. Another four samples were obtained from patients with CC who underwent concurrent chemoradiotherapy. These data were downloaded from GSE168009. In addition, batch sequencing data and clinical information of CESC patients have all undergone logarithmic conversion to achieve standardized data formats.

### 2.2. Analysis With Single‐Cell Sequencing Data

Before formal analysis, we used the R package “Seurat” to conduct quality control and preprocessing on the raw data [15]. Cells with less than 300 or more than 5000 detected genes were excluded. The “NormalizeData” function was applied to normalize data and convert scRNA‐seq data into a Seurat object [16]. In order to decrease the dimensionality of the data, the “RunPCA” function of the “Seurat” package was used for principal component analysis (PCA); then, the first 20 components were used for t‐SNE analysis. Subsequently, we used the “FindCluster” function for clustering and illustrating the results using UMAP [17]. “FindAllMarkers” was applied to find marker genes for different cell clusters. By utilizing classical marker genes, cell clusters in the generated two‐dimensional representation were annotated. The abundance of the sample was calculated by using the ssGSEA algorithm. ClusterGVis visualized gene expression patterns, clustering, and functional annotation.

### 2.3. Inferring Malignant Epithelial Cells

The R package “InferCNV” was applied to calculate the large‐scale chromosome copy number variation (CNV) of each somatic cell and differentiate normal cells from malignant epithelial cells based on the CNV score [18]. We used InferCNV to calculate CNV scores by averaging squared deviations from diploid state across genomic bins, with a classification threshold set at the mean + 3SD of reference normal cells. Then, we calculated sample abundance using the ssGSEA algorithm. After that, we used the Monocle2 algorithm for pseudotime analysis, with the gene cell matrix extracted from the Seurat subset and scaled UMI count as input [19]. CellChat is another important method to help understand the interactions between cells [20]. CellChat takes gene expression data of cells as input and simulates intercellular communication by combining the interactions of ligand receptors and their cofactors.

### 2.4. Gene Set Variation Analysis

GSVA is a classification way which can compare landmark functions and pathways across different groups. Fifty marker pathways from MSigDB were used to perform enrichment analysis. In order to give pathway estimates to each cell type, a comprehensive scoring of gene sets was conducted on each cell to assess potential biological functional changes in different samples [21]. Gene set with FDR < 0.05 was considered significantly enriched [22].

### 2.5. Establishment of Prognostic Model by Using Marker Genes

Firstly, we used the marker genes of Cluster 8 for univariate regression analysis to identify genes related to OS. Then, we screened them using LASSO analysis. After that, Cox analysis was employed to further select independent predictive genes and build a prognostic model in the training set. All the samples were assigned to two groups with the median score as the threshold. ROC validates the predictive accuracy of the CCM in the test set and all sets [23–25].

### 2.6. Mutation Situation

Tumor mutation can evaluate the production of tumor‐specific and highly immunogenic antibodies [26]. The frequency of somatic mutations and their distribution in a series of genes were analyzed using the “maftools” package. Then, patients were grouped on the base of median CCM scores and TMB values, and survival differences between these groups were examined.

### 2.7. Prediction of Tumor Microenvironment and Drug Sensitivity

Tumor cells and their microenvironment are a functional whole, and they interact with each other to promote the development of tumors [27]. Immunotherapy efficacy is related to TME. We used six algorithms to calculate the proportion of each immune cell. The differences in immune cell infiltration among these subgroups were visualized through heatmaps. Additionally, the “ESTIMATE” package was utilized to quantify the ESTIMATE score. To identify potentially effective chemotherapy drugs, the chemosensitivity of different groups was calculated using the “oncoPredict” package [28], and the “ggpubr” package was used to create box plots showing the differences.

### 2.8. Cell Line Culture

Human CC cell lines (HeLa and SiHa) were sourced from the Institute of Biochemistry and Cell Biology at the Chinese Academy of Sciences. The culture medium utilized was RPMI 1640, which was fortified with 10% fetal bovine serum (FBS) and 1% antibiotic solution, comprising 100 U/mL of penicillin and 100 mg/mL of streptomycin.

### 2.9. Cell Transfection and Lentivirus Construction

Lentiviral vectors encoding sequences for COL4A1 and short hairpin RNA (shRNA) were designed and synthesized by GenePharma, with the aim of modulating the expression levels of COL4A1 in CC cells. Once the cells reached a confluency of 50%, they underwent transfection with constructs incorporating COL4A1, control vector, shCOL4A1, and a negative control (sh‐NC). Following transfection, stable cell lines were selected using puromycin (Sigma‐Aldrich) over the course of 1 week. The efficiency of the transfection process was subsequently evaluated through qRT‐PCR analysis.

### 2.10. RNA Extraction and Quantitative Real‐Time PCR

mRNA underwent reverse transcription via the PrimeScript RT Reagent Kit (Takara). qRT‐PCR was conducted employing the TB Green Premix Ex Taq Kit (Takara). The relative expression levels of mRNA were normalized to GAPDH. The primer sequences are shown in Supporting Information 8: Table S1.

### 2.11. Colony Formation

A total of 200 cells were selected into each well of a 6‐well plate as part of the colony formation assay, and a standard growth medium was applied, which was subsequently replaced after a week. After allowing the colonies to mature over a 2‐week period, methanol was employed for 15 min, followed by staining with 0.1% crystal violet (Sigma) for a duration of 30 min. Upon completion of these steps, the resulting clones were counted to assess their colony‐forming efficiency.

### 2.12. Ethynyl Deoxyuridine (EdU)

The processes of labeling and staining with EdU were performed using an EdU cell proliferation detection kit sourced from RiboBio. Following the introduction of cells into 96‐well plates at a density of 5 × 103 cells per well, a 50 *μ*M EdU labeling medium was applied 48 h after transfection. The cells were then incubated at 37°C in a controlled environment with 5% CO_2_ for a duration of 2 h. Subsequently, the cells underwent treatment with 4% paraformaldehyde and 0.5% Triton X‐100 for the staining of the anti‐EdU working solution. Nuclei were stained using diamidino‐2‐phenylindole (DAPI). The evaluation of the proportion of EdU‐positive cells was conducted through fluorescence microscopy.

### 2.13. Migration and Invasion Assays

Migration and invasion assays were performed using the Transwell system, which consists of 24 wells with an 8 mm pore diameter. For the migration assays, a cell population of 5 × 104, following transfection, was placed in the upper chambers of the plates containing 350 *μ*L of serum‐free medium, while the lower chambers received 700 *μ*L of medium supplemented with 10% FBS. The Matrigel invasion assays involved the utilization of Transwell membranes that had been precoated with Matrigel (Sigma‐Aldrich). After a 16‐h incubation period, the cells on the upper surface were removed, and those were stained using methanol and 0.1% crystal violet.

### 2.14. Statistical Analysis

All statistical analyses were implemented using R 4.1.0. Kaplan–Meier was used to estimate and compare the subtype′s overall survival. The correlation between variables was evaluated through Pearson correlation analysis. *p* < 0.05 is considered statistically significant.

## 3. Results

### 3.1. The ScRNA Profiling of Samples

All 23 samples showed relatively stable cell distribution, laying a solid foundation for subsequent analysis (Figure 1a). Cell type identification was shown in Figure 1b. The distribution ratios of epithelial cells and seven immune‐related cell types in 23 samples are shown in Figure 1c. Among them, fibroblast cells and NK/T cells have a relatively high proportion. Figure 1d,e reveals the presence of four cell types in epithelial cells: normal clinical stage, early stage, late stage, and high‐grade cervical intraepithelial neoplasia.

Figure 1Basic information of the samples. (a) Distribution of cells in each sample. (b) The UMAP plot shows the expression of marker genes in different cell types. (c) The proportion of eight cell types derived from different samples. (d, e) Cell annotation reveals four disease stages.

**Figure:**
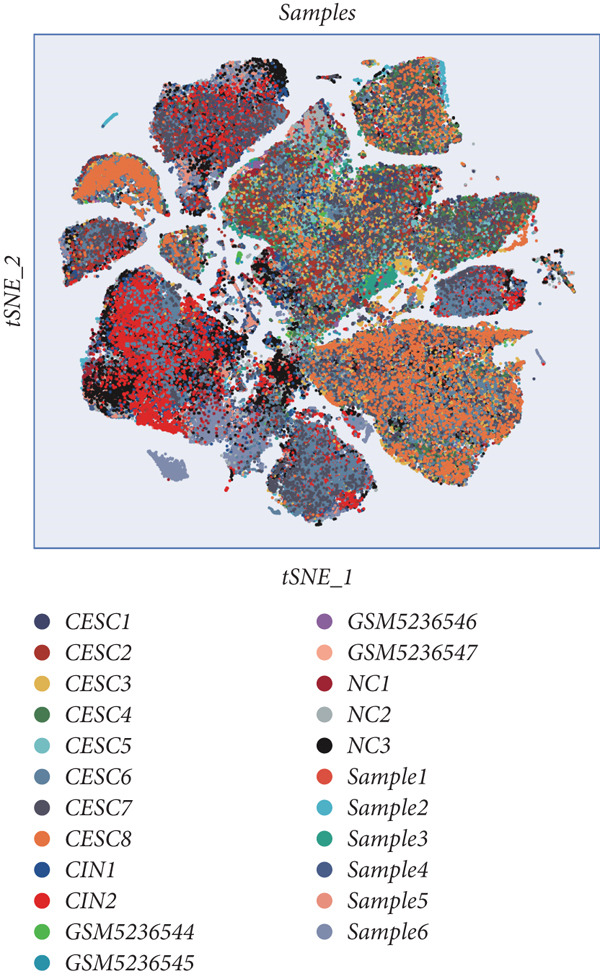
(a)

**Figure:**
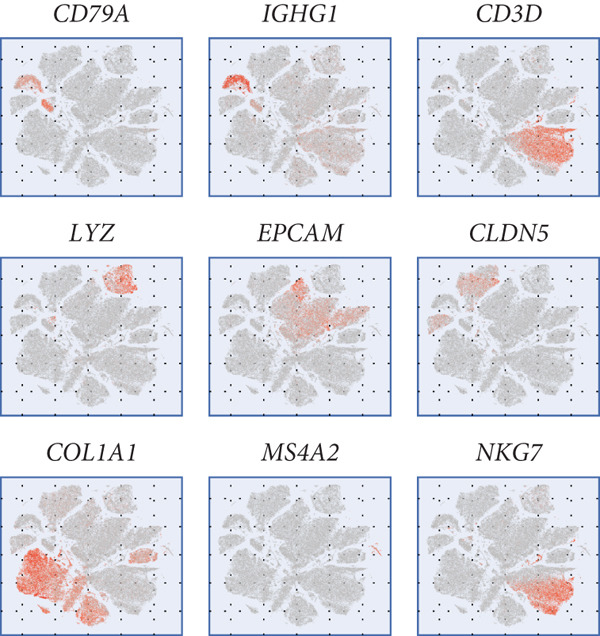
(b)

**Figure:**
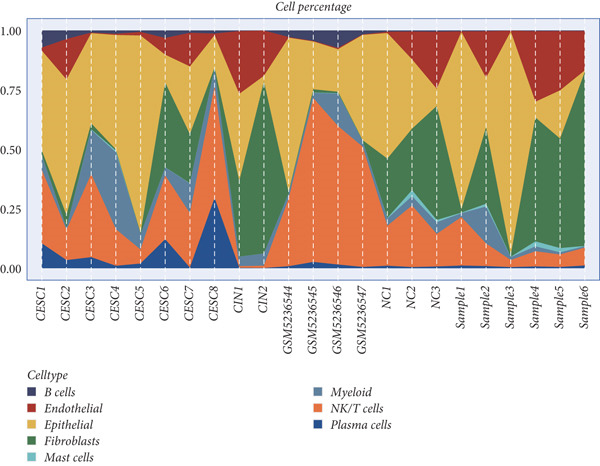
(c)

**Figure:**
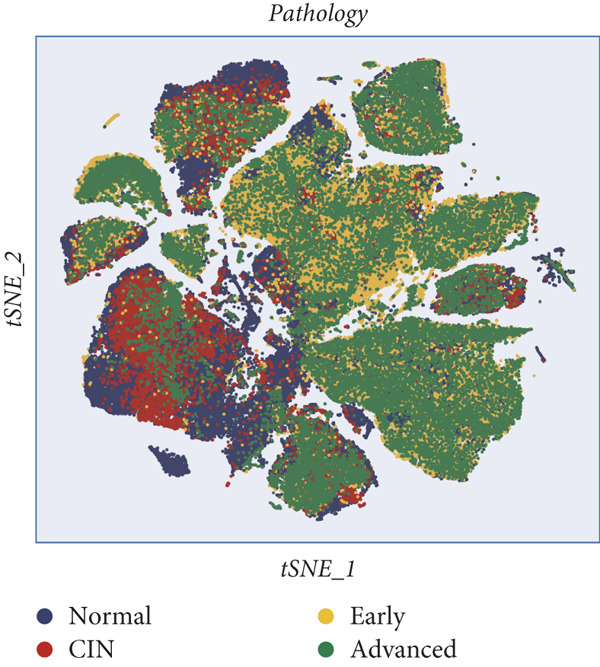
(d)

**Figure:**
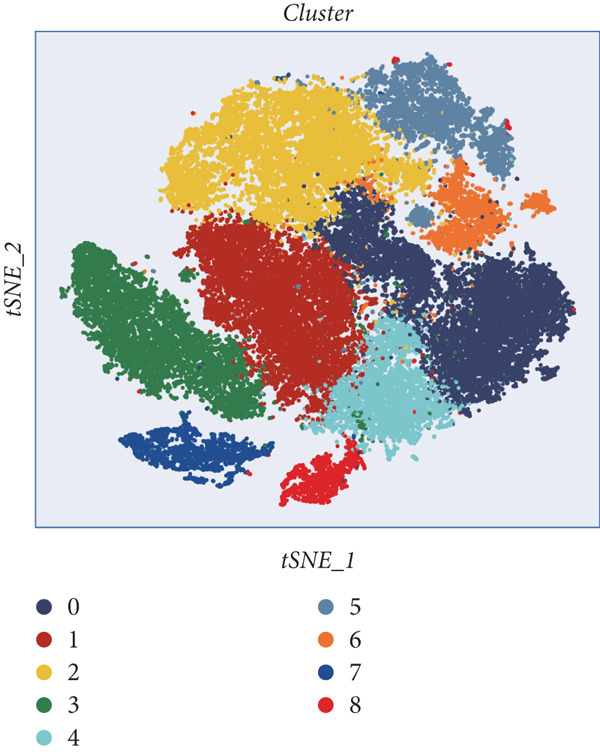
(e)

### 3.2. Explanation of Cell Subclusters

The epithelial cells were further divided into nine clusters (Figure 2a), and the corresponding proportions are shown in Figure 2b. It can be seen from the graph that the abundance of Cluster 8 decreases significantly with the progression of the disease (Figure 2c). Then, we calculated the copy number loss or amplification of each chromosome in epithelial cells, where Cluster 8 had a lower CNV score (Supporting Information 1: Figure S1), indicating a lower degree of tumor malignancy [29, 30]. Copy variation of each chromosome in epithelial cells is shown in Supporting Information 1: Figure S1. Samples with higher Cluster 8 abundance had better survival outcomes (Figure 2d), while other clusters had no influence (Supporting Information 2: Figure S2). We further explored the expression and changes of the differential genes in each cluster, as well as the functions of the genes, as shown in Figure 2e. Most of these functions are related to immunity. We then performed functional enrichment on nine clusters separately (Figure 2f). Cluster 8 was mainly enriched in epithelial–mesenchymal transition (EMT) and angiogenesis‐related pathways.

Figure 2Explanation of cell subclusters. (a) Cell annotation revealed nine different cell clusters. (b) The proportion of nine clusters. (c) Comparison of CNV ratings for nine clusters. (d) Survival curve of groups with different Cluster 8 abundances. (e) Heatmap simultaneously displays the gene expression and their functions. (f) Functional enrichment analysis of nine clusters.

**Figure:**
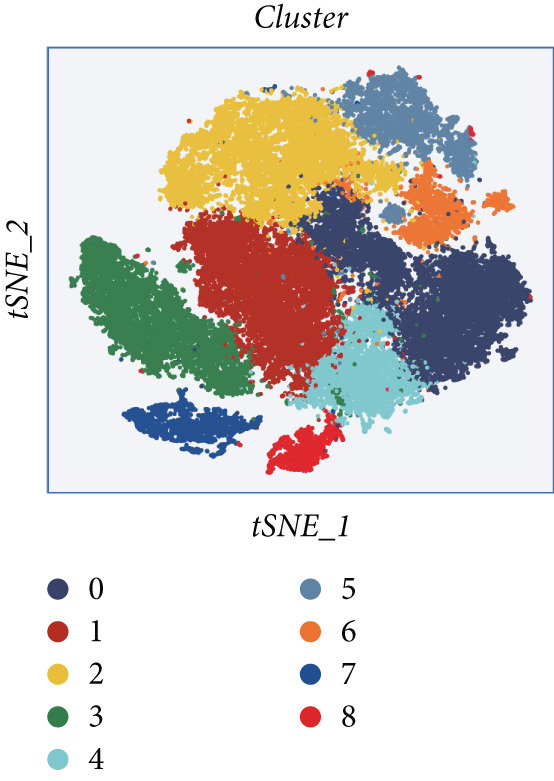
(a)

**Figure:**
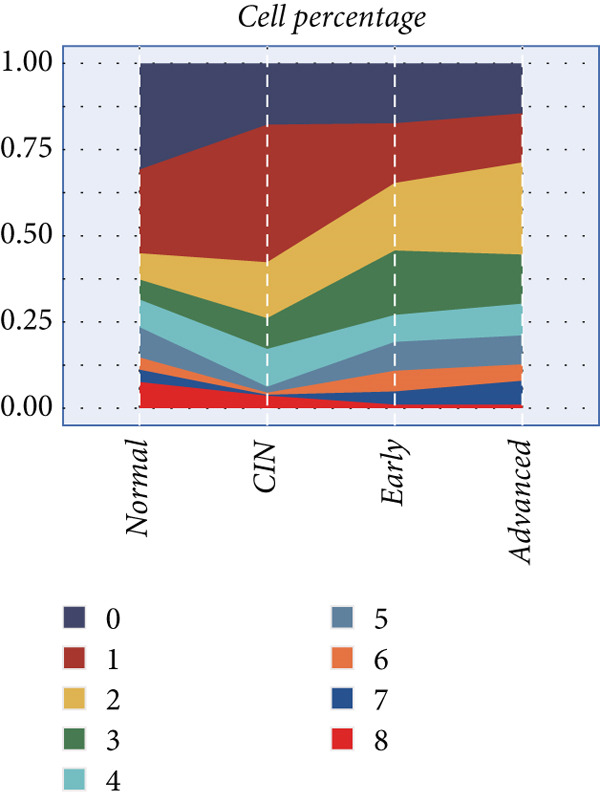
(b)

**Figure:**
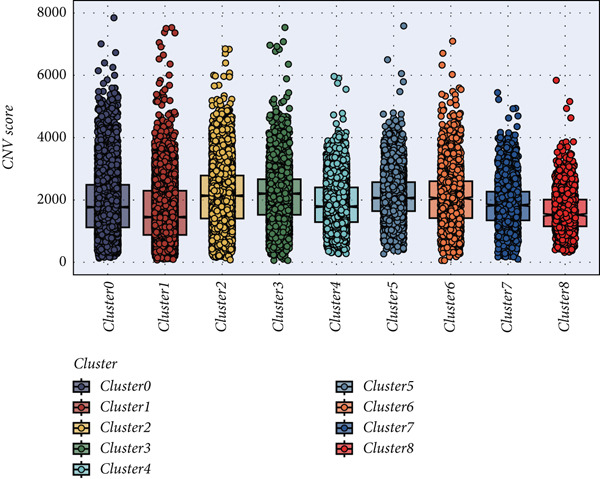
(c)

**Figure:**
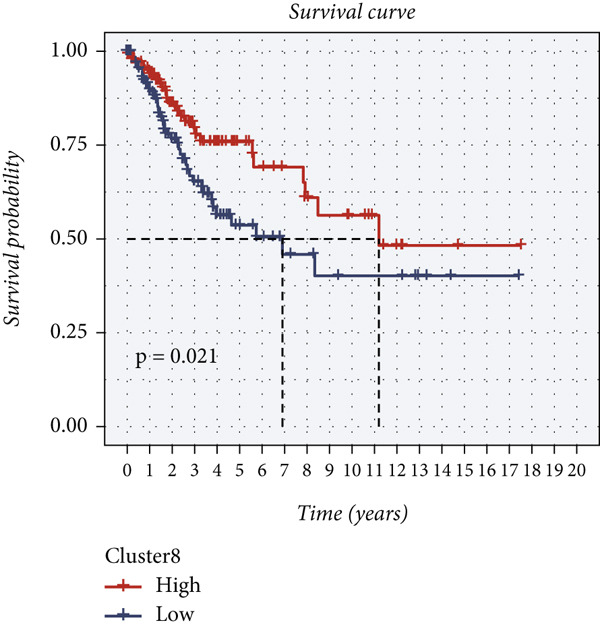
(d)

**Figure:**
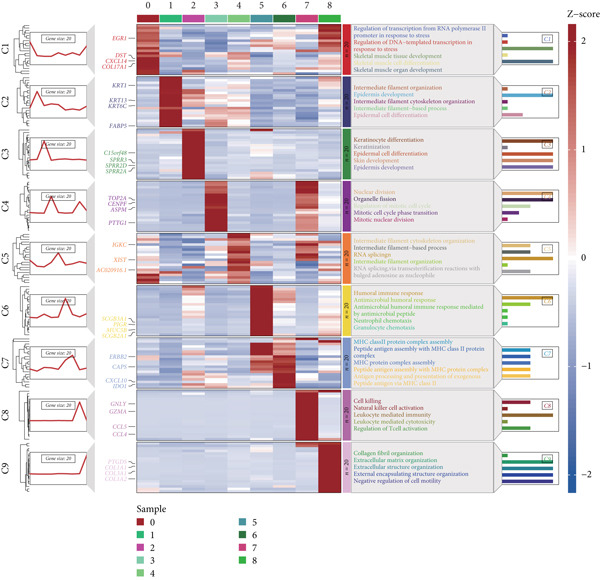
(e)

**Figure:**
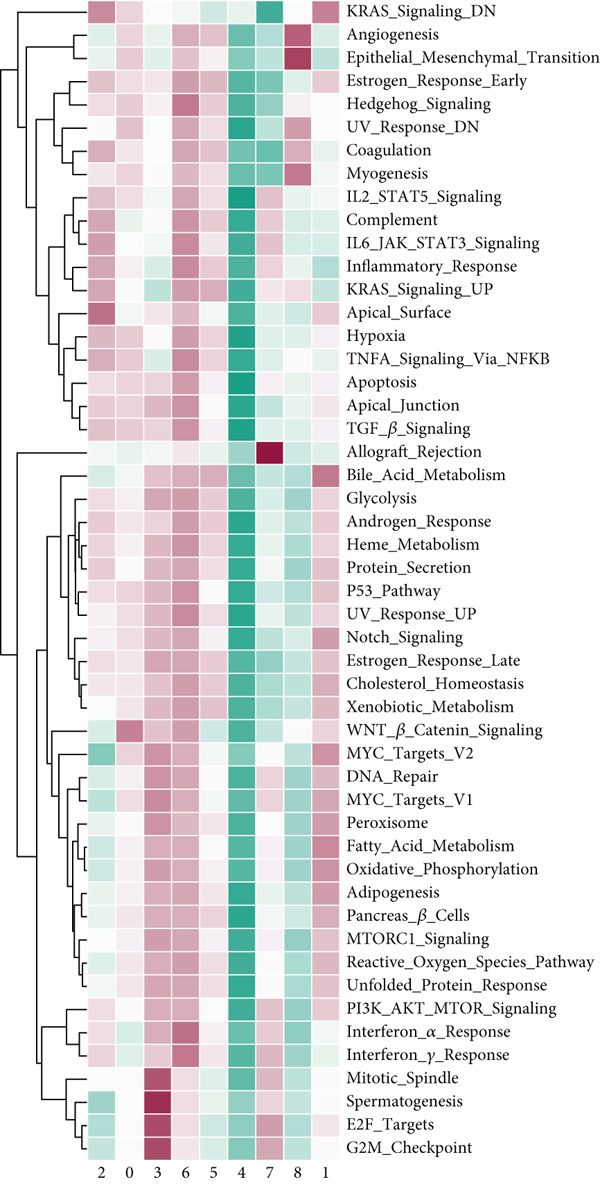
(f)

### 3.3. The Evolution and Interaction Between Different Clusters

The interactions and developmental trajectories between cells are of concern to us. The results show that Cluster 8 sends a signal to fibroblasts through the PTN signaling pathway (Figure 3a). PTN‐SDC2 and PTN‐SDC4 ligand–receptor pairs play important roles between Cluster 8 cells and fibroblasts (Figure 3b). This may be the reason for the extracellular matrix remodeling function in Cluster 8. Figure 3c shows the signals and their intensities emitted by Cluster 8 cells and fibroblasts as signal senders to other cell types. In the pseudotime inference analysis, a great enhancement happened in the proportion of Cluster 2 (Figures 3d, 3e, and 3f). Cell communication between other cell types is shown in Supporting Information 3: Figure S3A. In Supporting Information 3: Figure S3B, we analyzed ligand–receptor interactions between clusters and immune‐related cells. MDK‐NCL, MIF‐(CD74 + CXCR4), and MIF‐(CD74 + CD44) take great importance in multiple cell communications. Figure 3g illustrates that gene regulatory elements had differential expression in nine clusters and functional enrichment analysis was conducted on them (Supporting Information 4: Figure S4). It is enriched in some progress like cell death, extracellular vesicle, and protein binding.

Figure 3Cell communication analysis and pseudotime analysis. (a) Hierarchical plot showing cell communication between Cluster 8 and fibroblasts. (b) Cord plot of ligand–receptor pairs among the pituitary cell types. (c) The signals emitted by the signal sender to other cell types and their intensities. (d–f) All epithelial cells′ differentiation trajectories, pseudotime distribution, and cell clusters on pseudotime and the proportion of each cluster. (g) The distribution of gene regulatory elements in different clusters.

**Figure:**
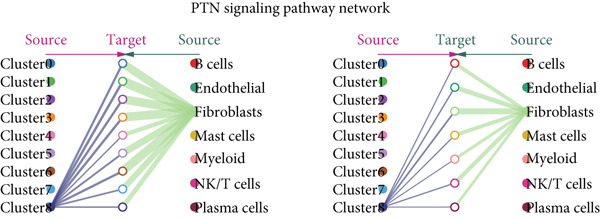
(a)

**Figure:**
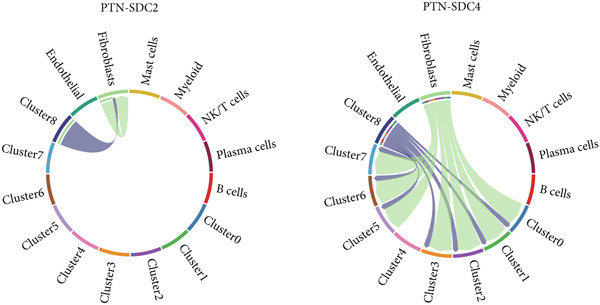
(b)

**Figure:**
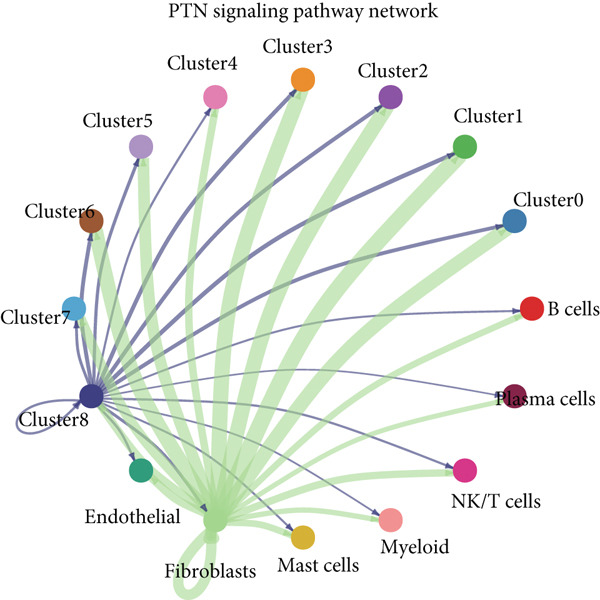
(c)

**Figure:**
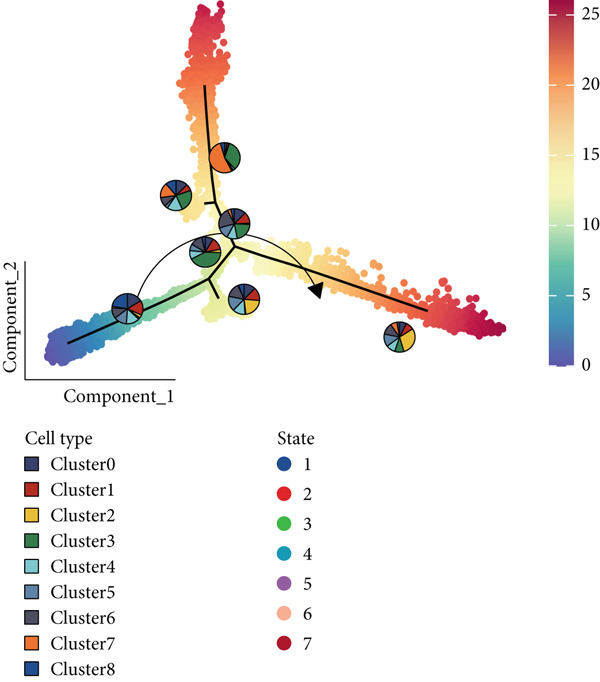
(d)

**Figure:**
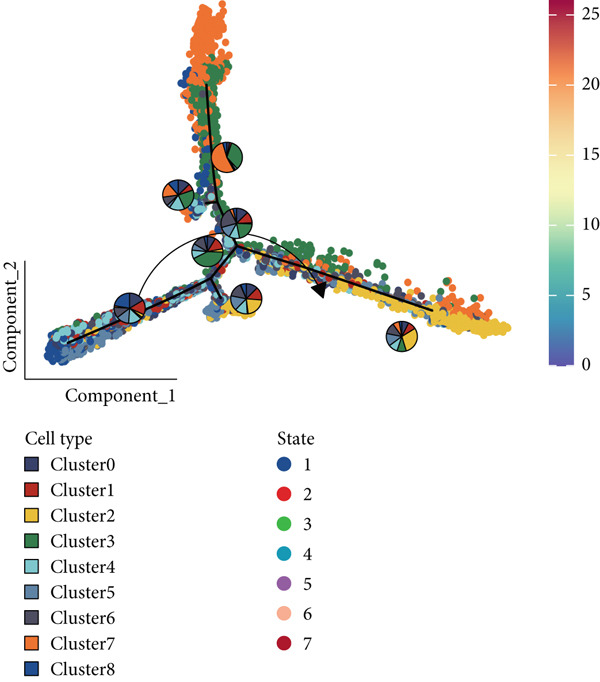
(e)

**Figure:**
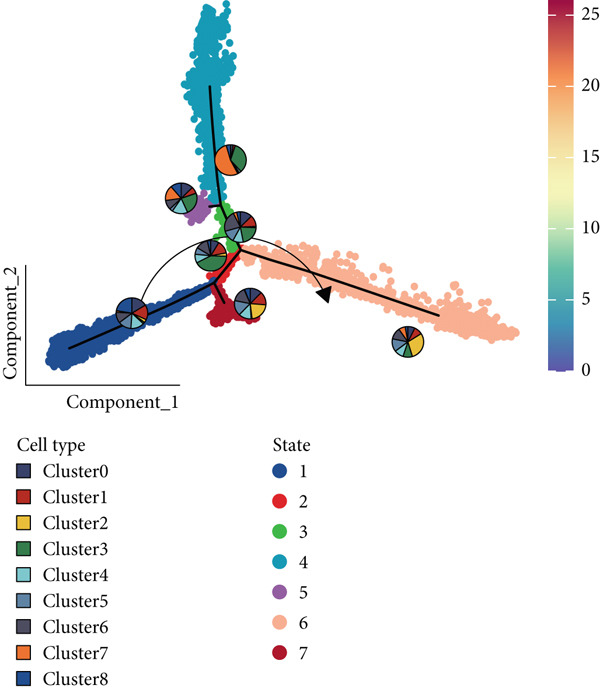
(f)

**Figure:**
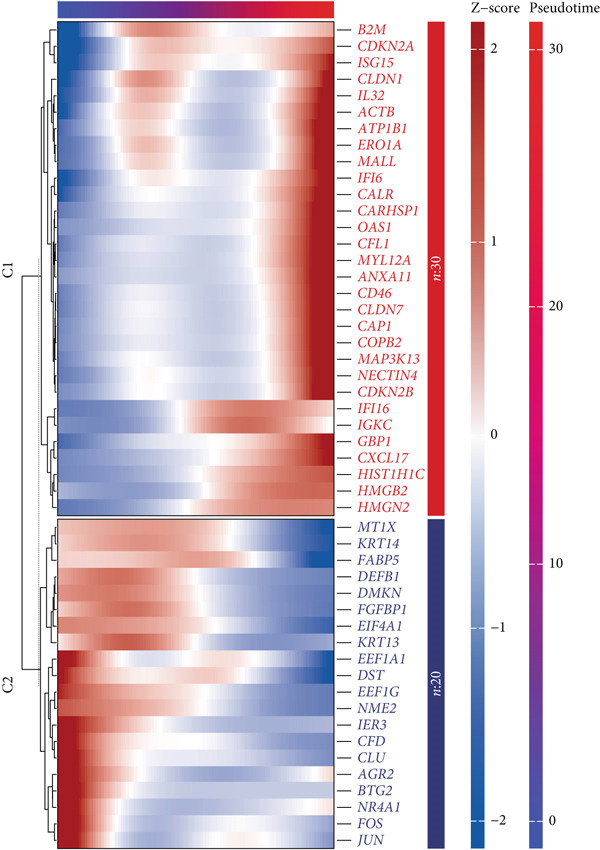
(g)

### 3.4. Establishing a Prognostic Model for CC

Marker genes in Cluster 8 were put into LASSO analysis to screen significant genes which can be applied to construct a prognostic model for CC (CCM) (Figure 4a). In light of the median CCM score, patients with scores higher than the median were considered high‐risk groups, while others were considered low‐risk groups. In the train set, the survival time of high‐risk group patients was lower, and the 5‐year predicted AUC could reach 0.8, indicating that the model has great prediction accuracy. This conclusion was validated in the test set and all sets (Figure 4b,c). After conducting PCA on all samples, it was found that high‐risk and low‐risk patients could be clearly distinguished (Figure 4d). This confirms the effectiveness of CCM again.

Figure 4(a) Screen predict signature through LASSO. (b, c) The survival curves of the two groups. (d) PCA analysis of three sets.

**Figure:**
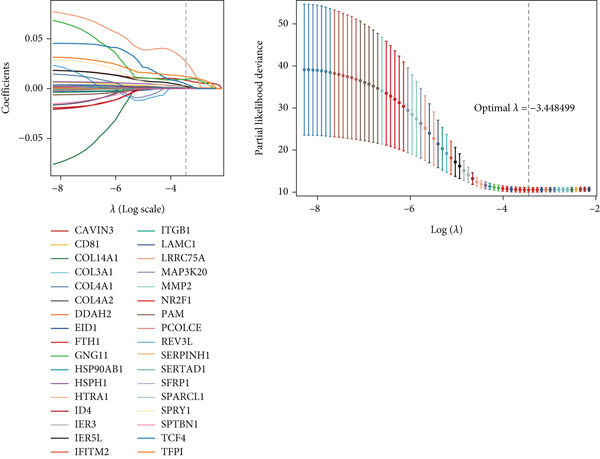
(a)

**Figure:**
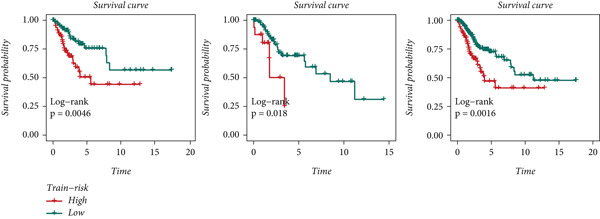
(b)

**Figure:**
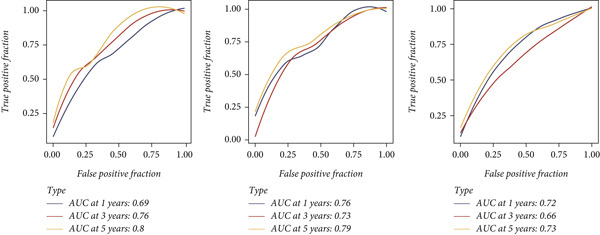
(c)

**Figure:**
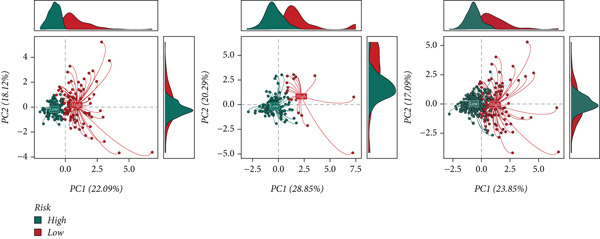
(d)

### 3.5. TMB and Immune Cell Infiltration

Distinct chromosomal alterations were frequently observed between the groups (Figure 5a). The specific altered genomic regions are shown in Figure 5b. There was a great difference in standardized TMB between the groups (Supporting Information 5: Figure S5). In light of the CCM score and TMB, further stratification of patients was conducted. We observed that the survival time of the low TMB group was lower than that of the high TMB group. People with lower TMB and higher CCM score had the worst prognosis (Figure 5c,d). Six algorithms were applied to infer the degree of immune cell infiltration (Figure 6a). There are great differences in ESTIMATE scores and tumor purity between the groups (Figure 6b). Immune cell infiltration is more pronounced in the low‐risk score group. There is a negative relationship between the CCM score and the ESTIMATE score. On the contrary, there is a positive association with CCM score and tumor purity (Supporting Information 6: Figure S6).

Figure 5Status of tumor mutation burden. (a) Chromosome amplifications and deletions. (b) Genomic alteration landscape in two risk groups. (c, d) Survival curves of survival differences between different subgroups.

**Figure:**
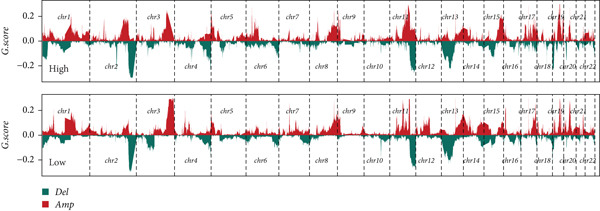
(a)

**Figure:**
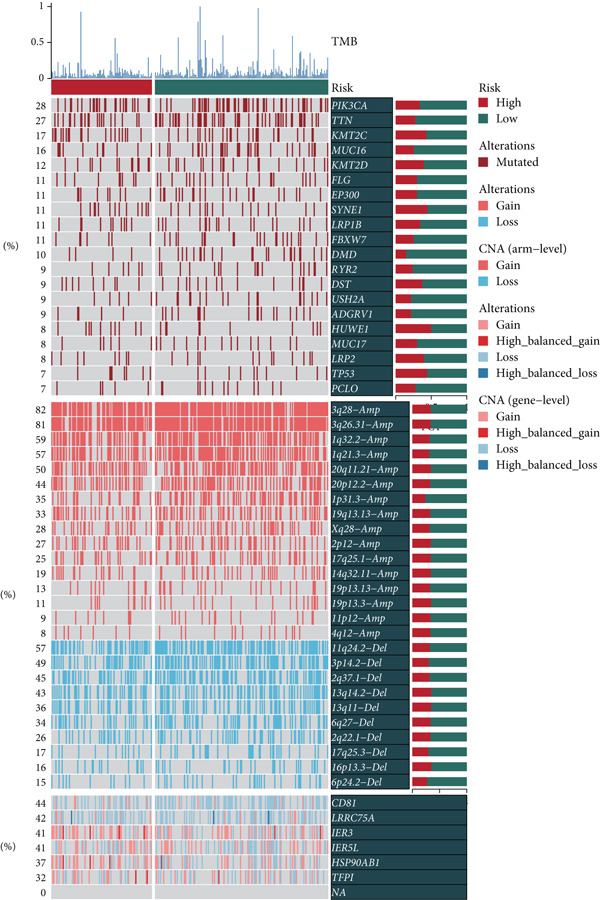
(b)

**Figure:**
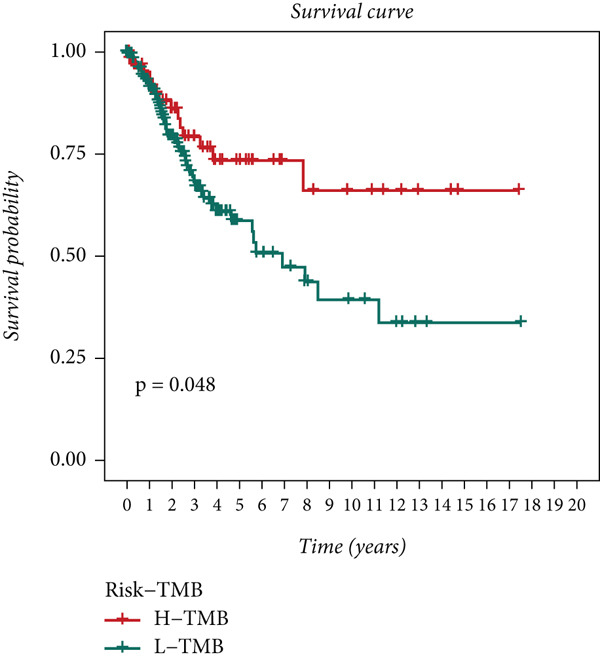
(c)

**Figure:**
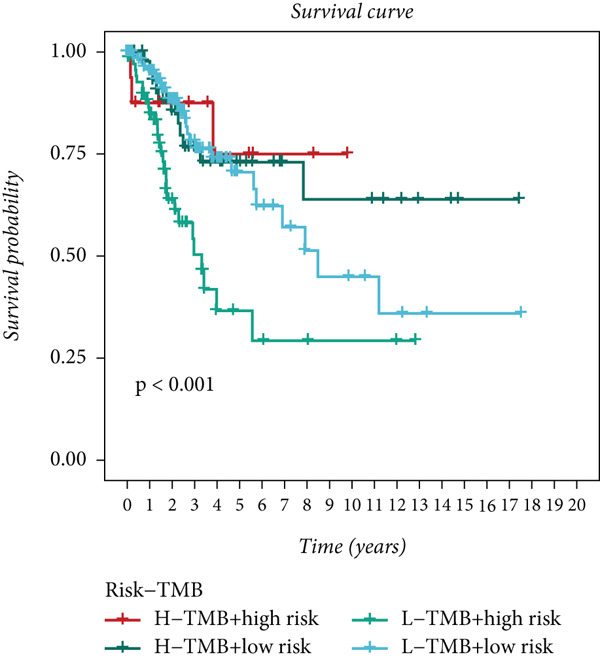
(d)

Figure 6Immune infiltration assessment. (a) The heatmap displays the immune cell infiltration between the groups. (b–e) Differences in tumor purity, ESTIMATE score, immune score, and stromal score between the groups.

**Figure:**
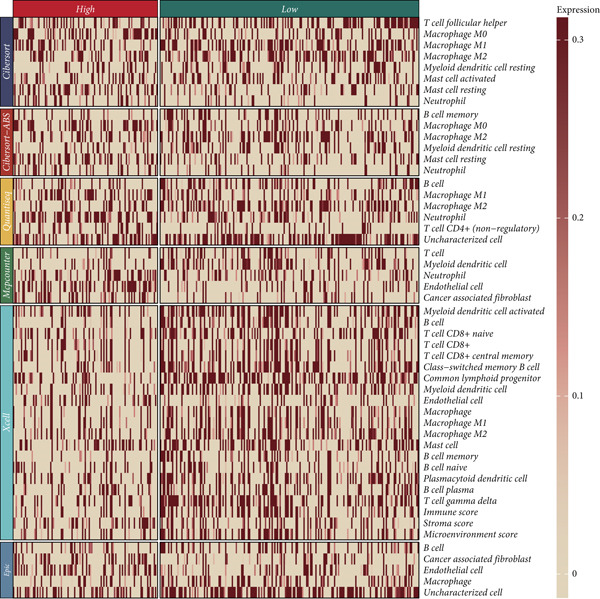
(a)

**Figure:**
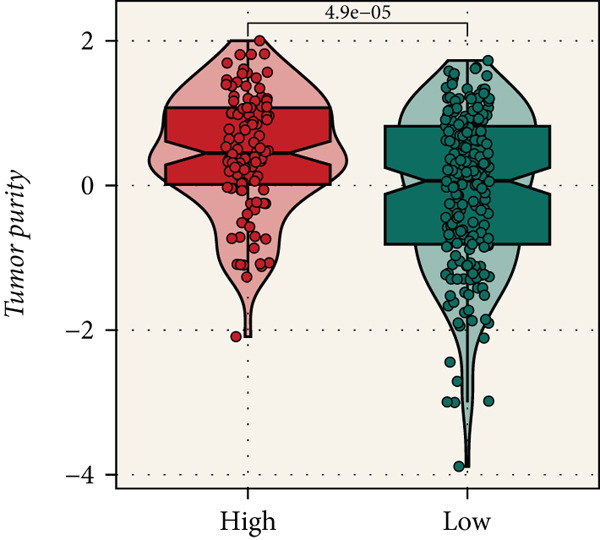
(b)

**Figure:**
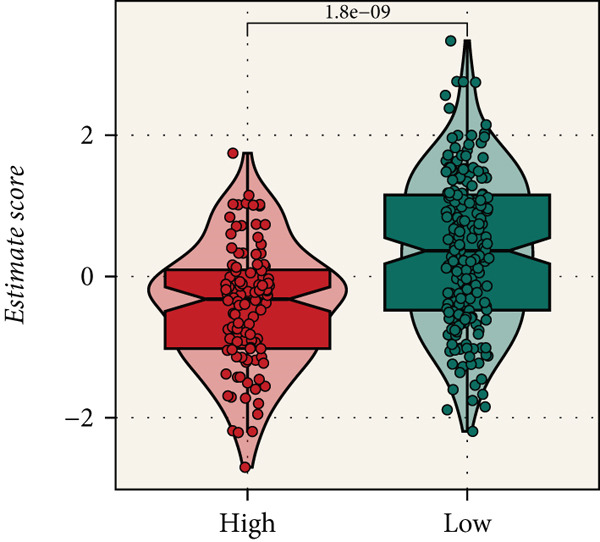
(c)

**Figure:**
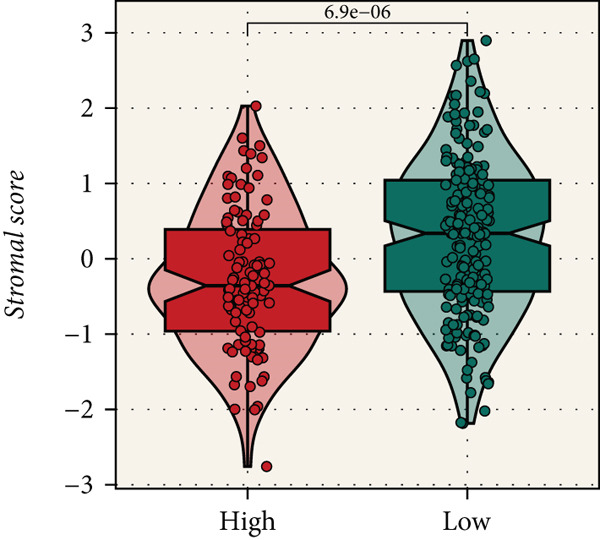
(d)

**Figure:**
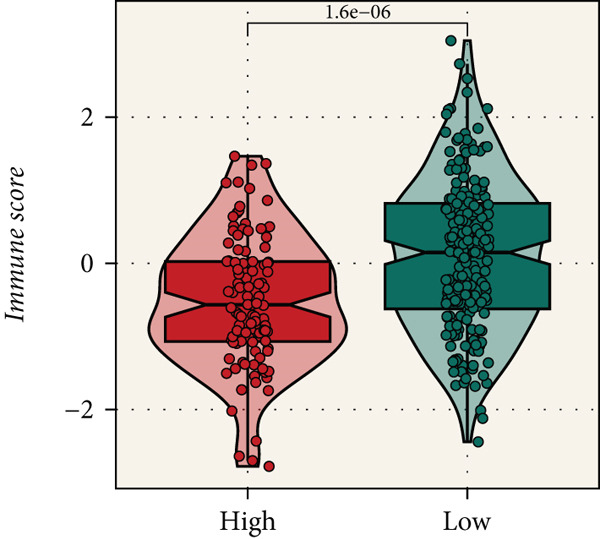
(e)

### 3.6. Analysis of Immune Cycle and Drug Sensitivity

We carried out an analysis on the association between the hallmark gene sets and immune cycle steps, revealing a negative correlation between CCM scores and immune cycles. However, there is a positive association between CCM scores and specific oncogenic pathways, including DNA repair and E2F targets (Figure 7a). These pathways are closely related to tumor progression. For example, DNA repair signaling pathways affect genome stability, and germline aberrations of key DNA repair genes can lead to cancer susceptibility DNA [31]. E2F plays crucial roles in cell proliferation and tumor suppression as the principal target of the tumor suppressor pRB [32]. KEGG analysis showed that the high‐risk group was related to the MAPK signaling pathway and regulation of the actin cytoskeleton. In contrast, melanoma‐related pathways were observed to be enriched in the low‐risk group. In addition, GO enrichment emphasized the enrichment of methyl‐CpG binding and pancreatic development in the high‐risk group, while fibroblast growth factor receptor signaling and voltage‐gated potassium channel activity were enriched in the low‐risk group (Figure 7b). Chemotherapy is a common method for treating tumors, and we have conducted research on potentially effective chemotherapy drugs for different subgroups. The results identified nine drugs, namely, PCI‐34051, AZD1332, sapitinib, pyridostatin, IWP‐2, cisplatin, AZD6482, AZD5991, and AZD3759, which may achieve better therapeutic effects when applied to low‐risk patients (Supporting Information 7: Figure S7).

Figure 7Immunotherapy analysis and enrichment analysis. (a) Association between hallmark gene sets and immune cycle steps. (b) GSEA shows enrichment of different genes.

**Figure:**
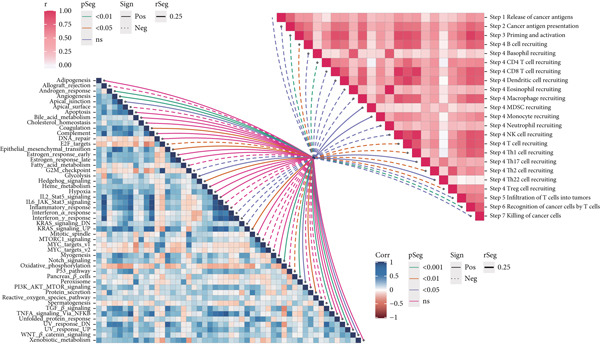
(a)

**Figure:**
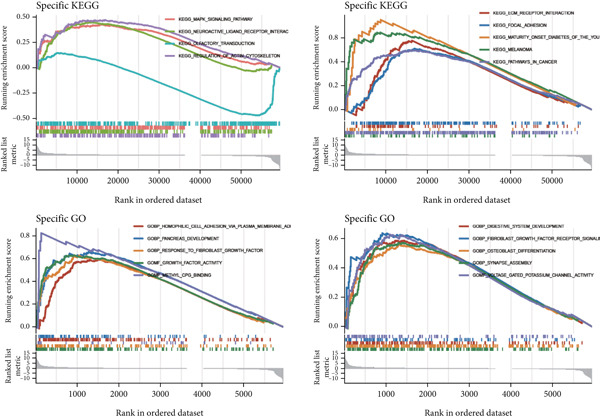
(b)

### 3.7. Experimental Verification of COL4A1

COL4A1 was chosen from our proposed signature for experimental validation, considering that it is currently little studied in CC. The results of PCR demonstrated good transfection efficiency in CC cell lines (Figure 8a). Since the knockdown effect of shCOL4A1‐1 and shCOL4A1‐2 resulted in the most pronounced decrease in the level of COL4A1 mRNA, we selected them for subsequent experiments. CCK‐8 assays showed that silencing COL4A1 greatly suppressed the cell proliferation in SiHa cells, while overexpression of COL4A1 significantly promoted the cell viability in HeLa cells (Figure 8b). Moreover, the clonality of CC cells was inhibited after silencing COL4A1, whereas increased by COL4A1 upregulation (Figure 8c,d). Similar results were obtained from the results of EdU assays (Figure 8c,d). Then, we explored the role of COL4A1 in CC cell metastasis by Transwell assays. As depicted in Figure 8e,f, knockdown of COL4A1 markedly blocked the migration and invasion of CC cells, whereas upregulation of COL4A1 significantly promoted CC metastasis.

Figure 8The effect of COL4A1 on proliferation and metastasis in CC cells. (a) The COL4A1 expression was detected after transfection in HeLa and SiHa cells. (b) CCK‐8 assays with COL4A1 knockdown or overexpression, respectively. (c, d) The role of COL4A1 on proliferation in CC cells was determined by colony formation assay and EdU assay. (e, f) The migration and invasion capacities of transfected CC cells.  ^∗∗^ *p* < 0.01;  ^∗∗∗^ *p* < 0.001. Scale bar, 200 *μ*m.

**Figure:**
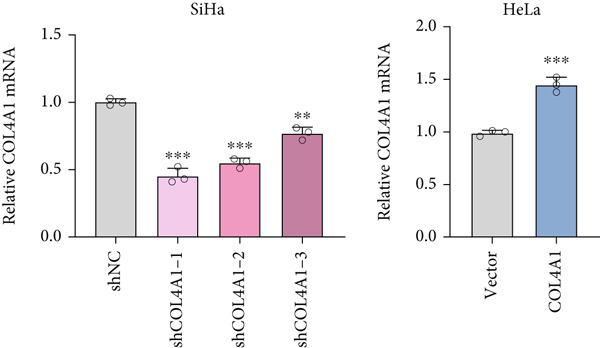
(a)

**Figure:**
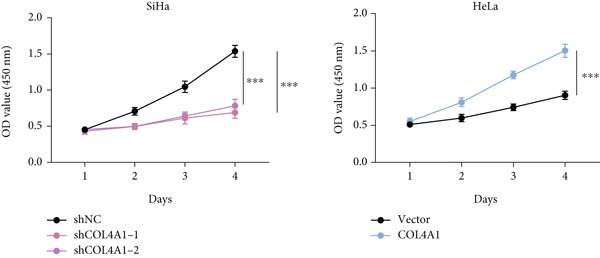
(b)

**Figure:**
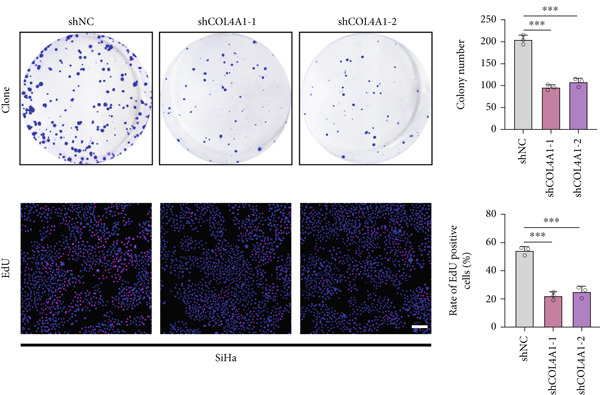
(c)

**Figure:**
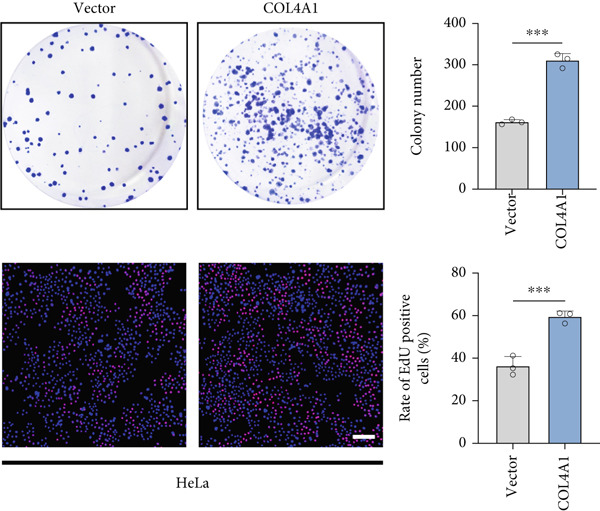
(d)

**Figure:**
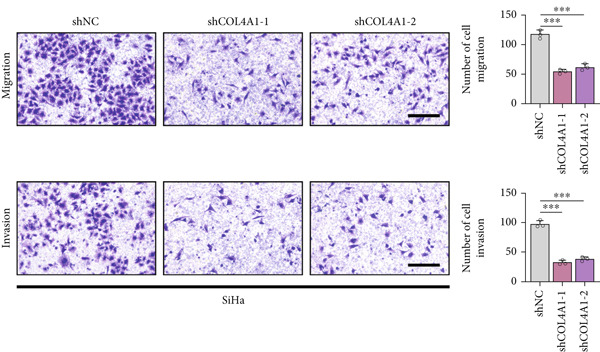
(e)

**Figure:**
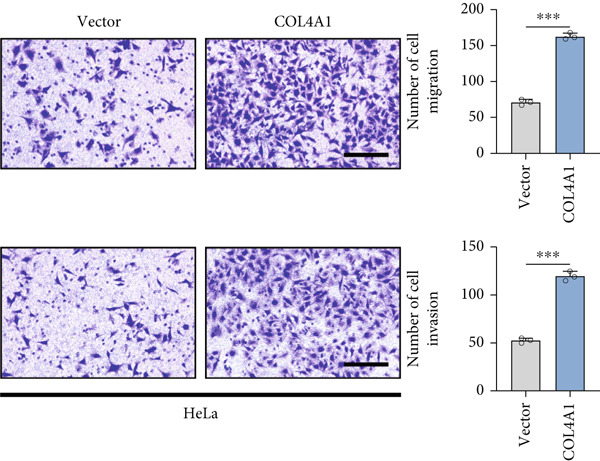
(f)

## 4. Discussion

CC is a public health problem that cannot be ignored at present. Although the human oncovirus vaccine has been widely used, the incidence rate and mortality of CC are still on the rise [33]. Due to the strong metastasis and proliferation of CC cells, patients have a poor postoperative prognosis and a low 5‐year survival rate. Therefore, we urgently need to further explore the molecular mechanisms underlying the occurrence and development of CC and search for new therapeutic targets.

In this article, we used scRNA‐seq to depict the developmental trajectory and interactions of human cervical constituent cells at single‐cell resolution, which will help understand the heterogeneity of cervical epithelial cells and intratumoral crosstalk. This level of analysis helps identify independent prognostic features of diseases. Single‐cell sequencing analysis also contributes to a deeper exploration of complex TMEs at single‐cell resolution, which may drive personalized choices for immunotherapy and drug therapy.

Revealing the cellular composition of CC is crucial for knowing its related immunological features. There is a high proportion of epithelial cells, fibroblasts, and NK/T cells in the cervical tissues of patients with CC. Epithelial tissue forms a structural barrier on the surface of human tissue that can resist microorganisms, environmental toxins, and mechanical stress [34]. The cells are capable of detecting various surface disturbances and then transmitting signals to the immune system to coordinate necessary immune cell responses. As an important component of tumor stroma, fibroblasts promote cancer cell proliferation and angiogenesis and induce drug resistance through immune regulation and fibrosis. Meanwhile, some studies suggest that CAFs can exert tumor‐suppressive effects in specific types of cancer [35]. NK cells are also essential innate lymphoid immune cells for tumor defense [36]. There is complex crosstalk between tumors and T cells, which can inhibit or promote tumor growth. It depends on the balance of the intricate interplay between them [37]. The function of these cells in the occurrence and progression of CC has not been fully clarified, and there is an urgent need for more research to investigate their potential as therapeutic targets for CC.

Among the nine identified epithelial cell subgroups, Cluster 8 is the only one related to survival, mainly enriched in EMT and angiogenesis. The activation of EMT is a potential cellular biological program involved in development, in which tumor cells undergo EMT to acquire malignant migration and stem cell characteristics. Angiogenesis means the sprouting and remodeling of new capillaries from pre‐existing functional blood vessels and is one of the fundamental biochemical pathways for the growth and metastasis of solid tumors. Using proangiogenic and antiangiogenic molecules as potential therapeutic drugs for this type of disease, it has a wide range of anticancer activities [38, 39]. Cluster 8 sends signals to fibroblasts through the PTN signaling pathway. Previous studies proved that PTN is elevated in both invasive nonmetastatic and metastatic diseases. CAF can also recruit immune cells to promote immune escape and inhibit immune cells to achieve immune tolerance [40]. Our research confirms that PTN‐SDC2 and PTN‐SDC4 ligand–receptor pairs act on fibroblasts, thereby promoting CC progression through the PTN signaling pathway.

Observing the TMB and CNV in the genome, the TMB of people with a higher CCM score was lower than that of people in the low‐risk group, proving that patients with a higher risk may have a higher burden of gene mutations. The higher the TMB, the easier it is for the immune system to detect cancer and activate defense mechanisms, and immune cells will search for and kill cancer cells. This is consistent with our research findings that the survival time of patients in the low TMB group is lower. Not surprisingly, in our study, patients with lower TMB and higher CCM scores had the worst prognosis. Single‐cell CNV is a direct manifestation of genomic instability in cells. Due to the disruption of genomic stability, cancer cells accumulate a large number of CNVs during proliferation. These abnormal CNVs may lead to the activation of proto‐oncogenes and the inactivation of tumor suppressor genes, thereby promoting tumorigenesis and progression [41].

Immunotherapy is generally considered an efficient treatment option for CC [42]; TMB has gained attention as a prospective biomarker for immunotherapy response [43, 44]. Low TMB tumors are believed to be “cold” tumor microenvironment, making them less responsive to ICB [45–47]. Higher TMB may promote immune cell infiltration in CC, making patients more likely to benefit from immunotherapy [48].

Immunotherapy is often combined with chemotherapy and radiation therapy [49]. Cisplatin is considered the standard treatment for metastatic CC [50], while about 30%–40% of such patients fail to achieve ideal results, and more effective alternative methods are needed. At present, researchers are studying novel drugs targeting molecular pathways. With an increasing number of available drugs, platinum‐based monotherapy has been retained as an alternative option under the “other recommended regimens.” The results identified nine drugs, namely, PCI‐34051, AZD1332, sapitinib, pyridostatin, cisplatin, AZD6482, AZD5991, and AZD3759, which may achieve better therapeutic effects when applied to low‐risk patients. Except for cisplatin, the role of other drugs in the treatment of CC is rarely mentioned. PCI‐34051 has been found to inhibit the growth of ovarian cancer and human bronchial smooth muscle cells as an inhibitor of HDAC8. Sapitinib can block the overexpression of ABCB1 transporter, which is effective for breast cancer cells and non–small cell lung carcinoma [51]. Pyridostatin inhibits cell growth by inducing replication and transcription‐dependent DNA damage [52]. AZD6482 can be used as an adjuvant drug for the treatment of glioblastoma [53]. AZD5991 binds to Mcl‐1 and induces cancer cell apoptosis, which has been applied in hematological malignancies [54–56]. The antitumor effect of AZD375 on non–small cell lung cancer has been confirmed [57, 58]. Although these drugs have varying degrees of anticancer effects, further research is needed to identify whether they can truly be used for the treatment of CC.

Nevertheless, our study has some limitations. Firstly, relying on 23 samples may limit the generalizability of our research findings. Then, scRNA‐seq may lose information about spatial location, which is not conducive to identifying the intercellular communication of cell types. In addition, both immune infiltration analysis and drug sensitivity analysis are based on algorithm analysis; more clinical trials are needed to prove our conclusions.

## 5. Conclusion

This study offers a comprehensive map of cellular characteristics of CC initiation and progression. In addition, we established a prognostic model of CC. It can identify individuals who would benefit from immunotherapy and chemotherapy which may promote the development of personalized treatment in clinical practice. Moreover, our results support the notion that COL4A1 could serve as a promising therapeutic target in the advancement of CC.

## Consent

The authors have nothing to report.

## Disclosure

All authors read and approved the final manuscript.

## Conflicts of Interest

The authors declare no conflicts of interest.

## Author Contributions

All authors contributed to the study conception and design. Conceptualization: J.L.; methodology: Y.S. and F.G.; formal analysis and investigation: R.G. and Z.Z.; writing—original draft preparation: R.G., Z.Z., and F.G.; writing—review and editing: R.J. and P.Z.; funding acquisition: J.L. and Y.S.; resources: J.L. and Y.S.; supervision: J.L. and P.Z. R.J., R.G., Z.Z., and F.G. contributed equally to this work.

## Funding

This work was funded by the Jiangsu Province Nature Science Foundation (No. BK20220729) and the Research Project of the Jiangsu Provincial Health Commission (Grant No. M2022078).

## Supporting Information

Additional supporting information can be found online in the Supporting Information section.

## Supporting information


**Supporting Information 1** Figure S1: Copy variation of each chromosome in epithelial cells.

**Supporting Information 2** Figure S2: The impact of the abundance of each cluster on survival.

**Supporting Information 3** Figure S3: The interaction between cell types and immune‐related cells. (A) Interaction net count plot of cells. (B) Bubble plots of different cell ligand receptors acting on cells.

**Supporting Information 4** Figure S4: Functional enrichment of gene regulatory elements.

**Supporting Information 5** Figure S5: Box plot of copy number variation differences.

**Supporting Information 6** Figure S6: Scatter plot illustrates the correlation between CCM score and matrix score, immune score, ESTIMATE score, and tumor purity.

**Supporting Information 7** Figure S7: Box plots compare the sensitivity of the groups to nine chemotherapy drugs.

**Supporting Information 8** Table S1: Sequences of qRT‐PCR primers or shRNA.

## Data Availability

The datasets used and/or analyzed during the current study are available from the corresponding authors on reasonable request. Data from the TCGA cohort and GEO are public.
