# Supplementary figures and images for: Exploring the Dynamic Changes of Intercellular Connections in Cervical Cancer: Insights From Transcriptomic Data Combined With Single‐Cell Sequencing
Source: Hum Mutat. 2026 Jan 22;2026:8140041. doi: 10.1155/humu/8140041 (PMC12828070; doi:10.1155/humu/8140041)

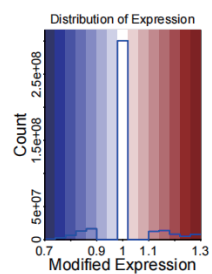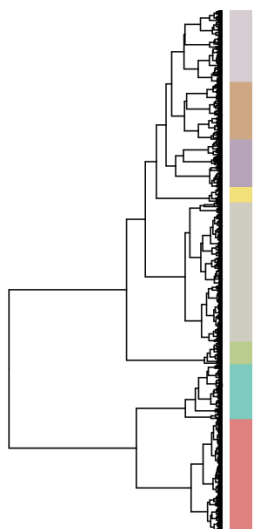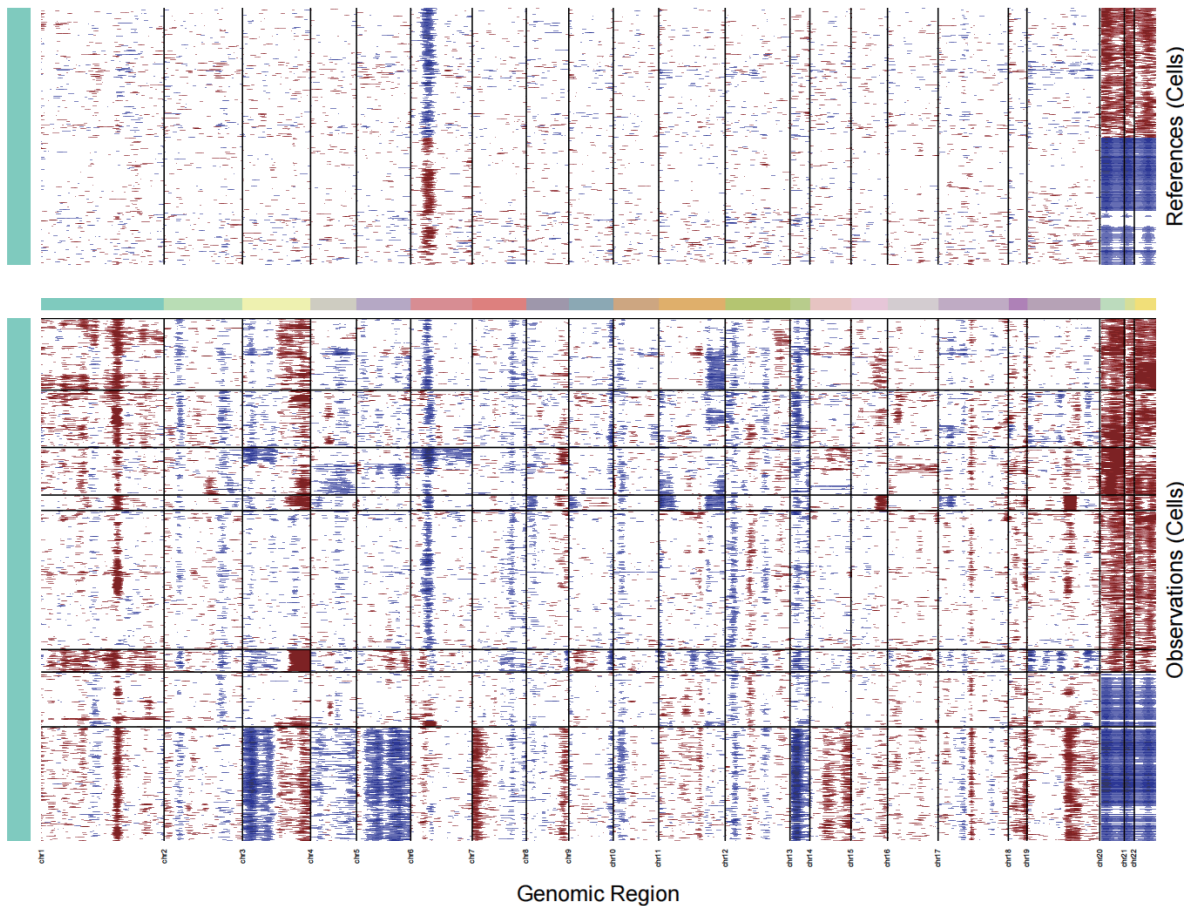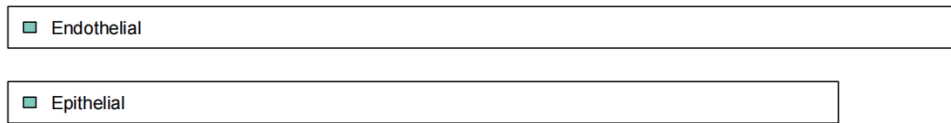

Supplement: Supplementary file 1 — Supporting Information 1 Figure S1: Copy variation of each chromosome in epithelial cells. [file HUMU-2026-8140041-s007.pdf]

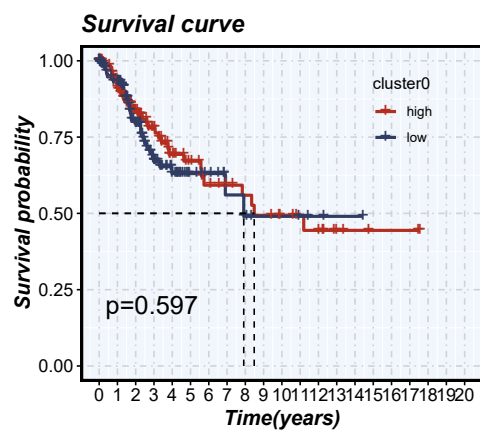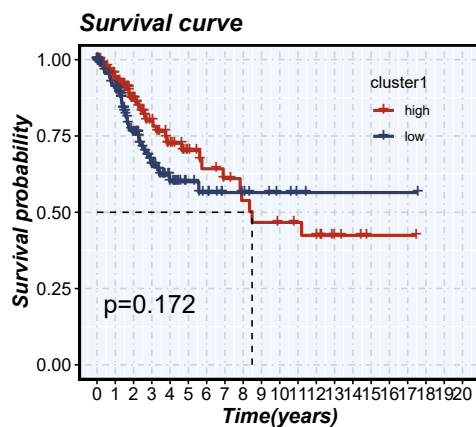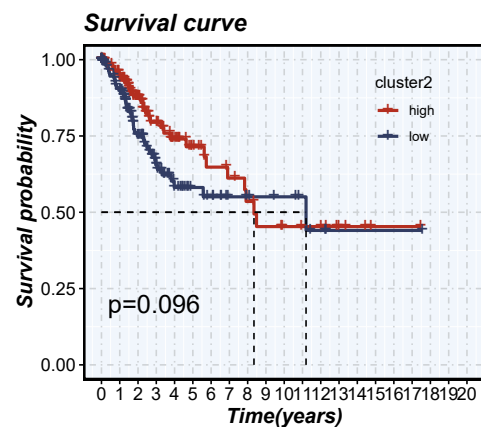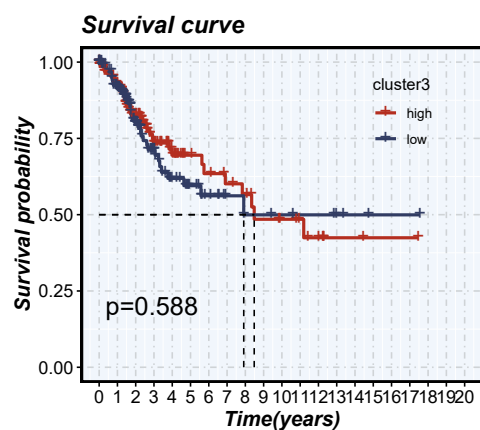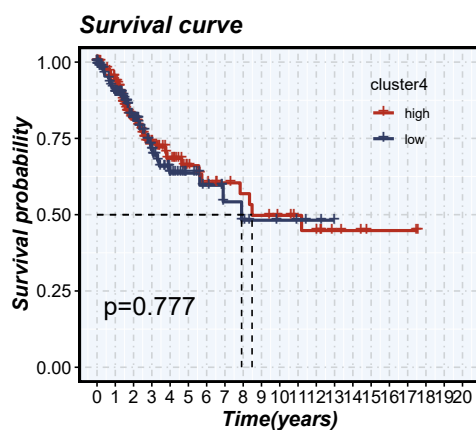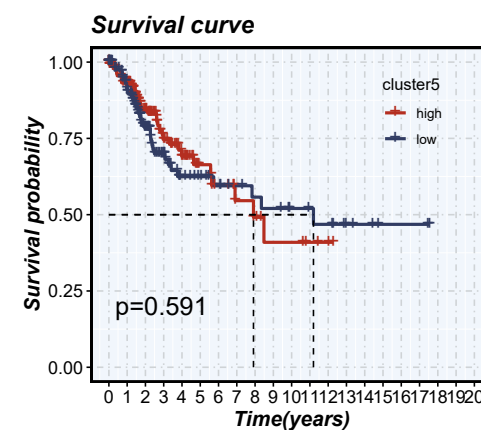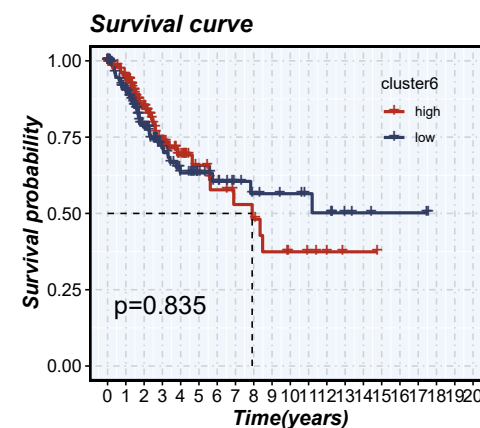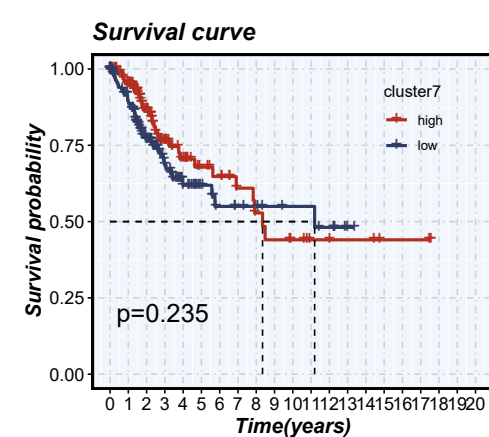

Supplement: Supplementary file 2 — Supporting Information 2 Figure S2: The impact of the abundance of each cluster on survival. [file HUMU-2026-8140041-s001.pdf]

A

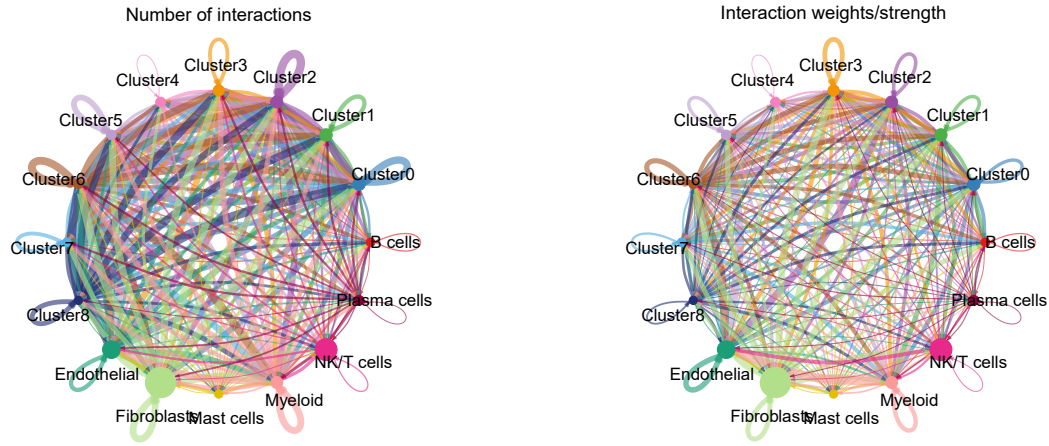

B

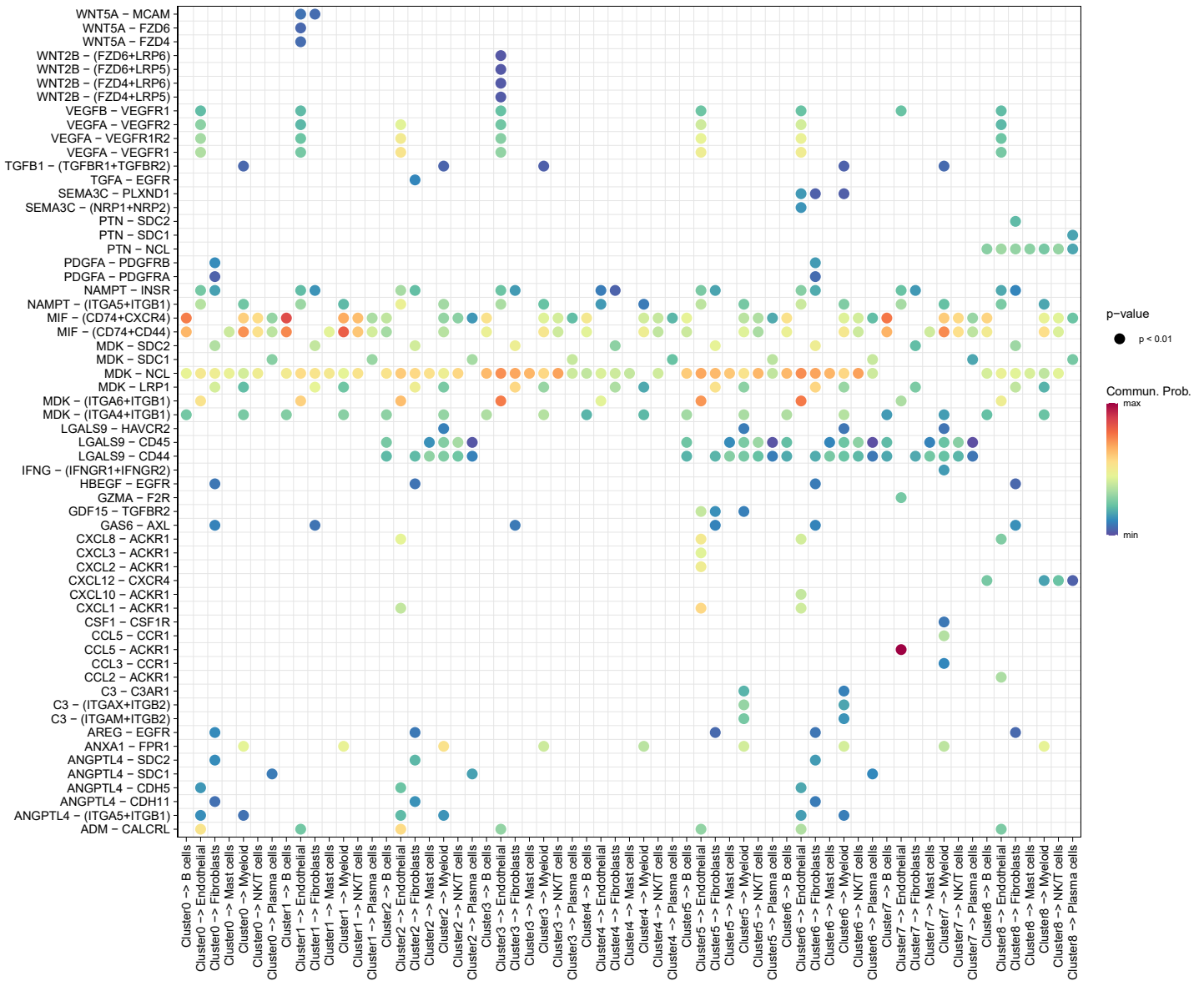

Supplement: Supplementary file 3 — Supporting Information 3 Figure S3: The interaction between cell types and immune‐related cells. (A) Interaction net count plot of cells. (B) Bubble plots of different cell ligand receptors acting on cells. [file HUMU-2026-8140041-s002.pdf]

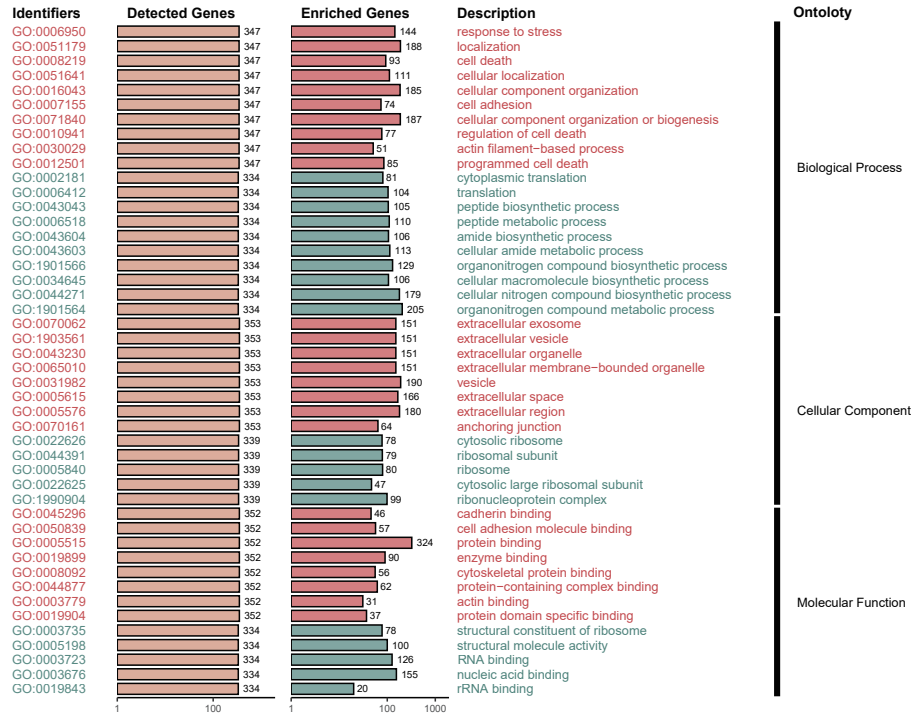

Supplement: Supplementary file 4 — Supporting Information 4 Figure S4: Functional enrichment of gene regulatory elements. [file HUMU-2026-8140041-s008.pdf]

A

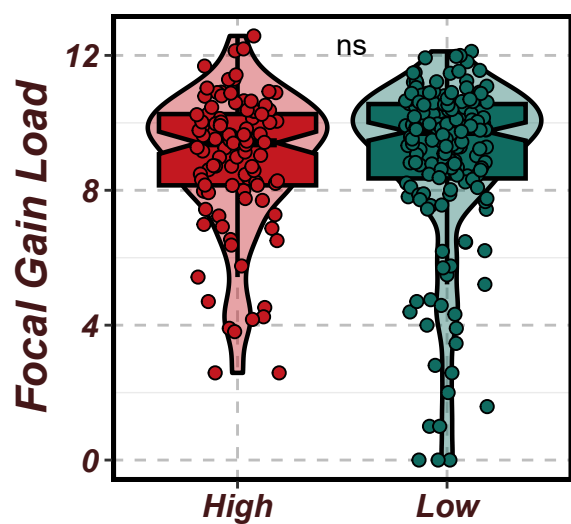

B

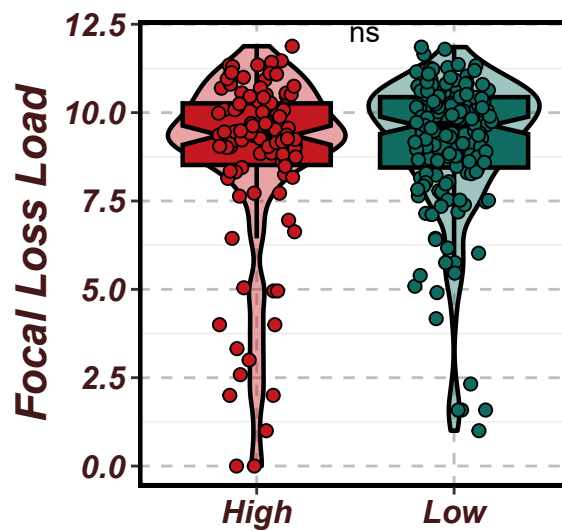

C

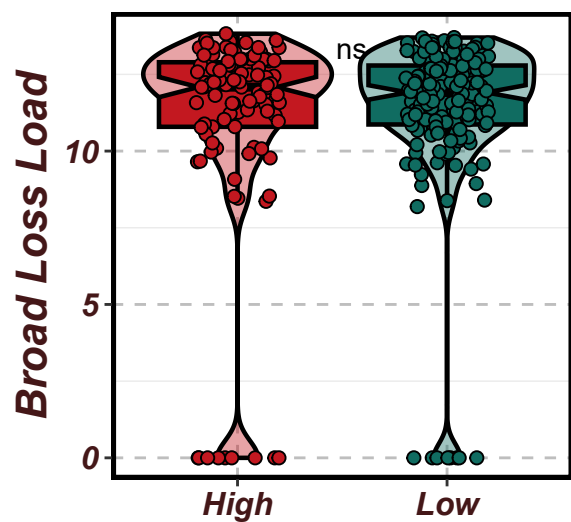

D

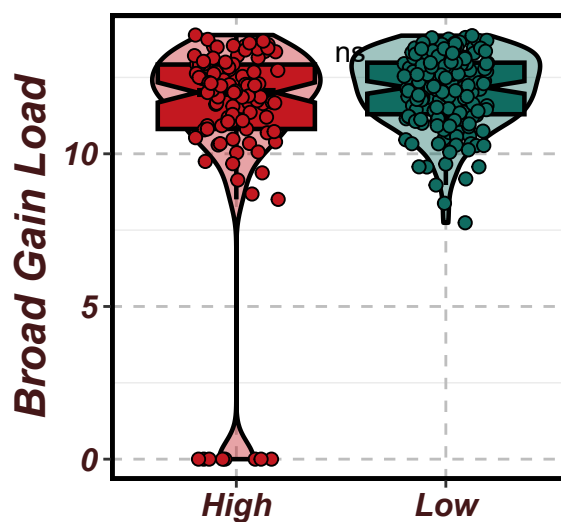

E

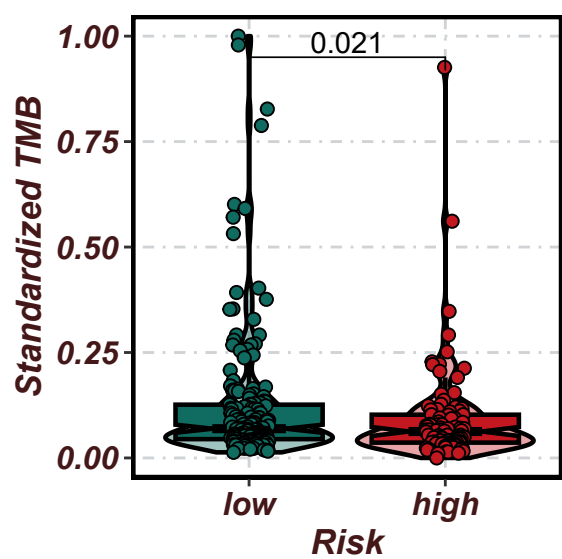

Supplement: Supplementary file 5 — Supporting Information 5 Figure S5: Box plot of copy number variation differences. [file HUMU-2026-8140041-s004.pdf]

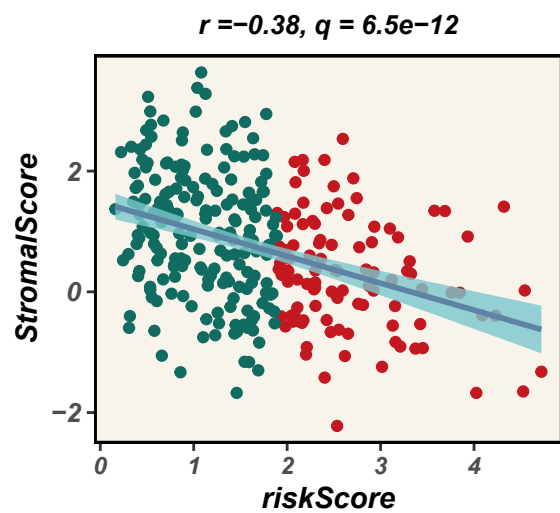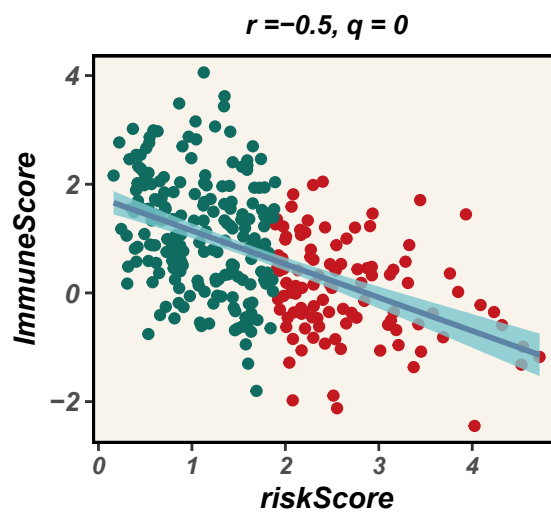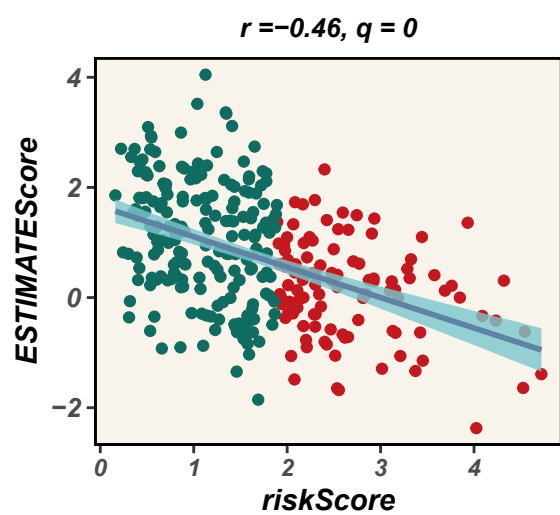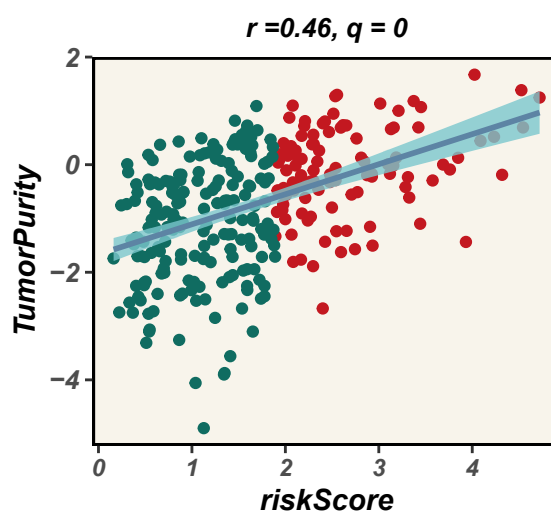

Supplement: Supplementary file 6 — Supporting Information 6 Figure S6: Scatter plot illustrates the correlation between CCM score and matrix score, immune score, ESTIMATE score, and tumor purity. [file HUMU-2026-8140041-s005.pdf]

A

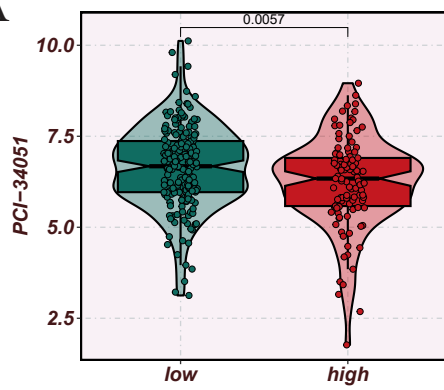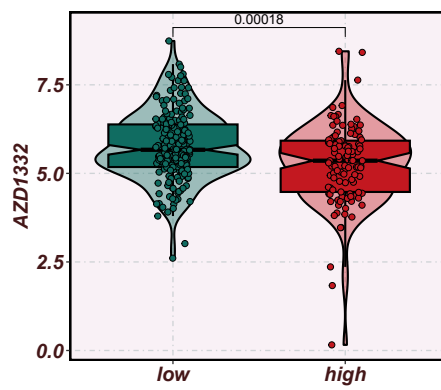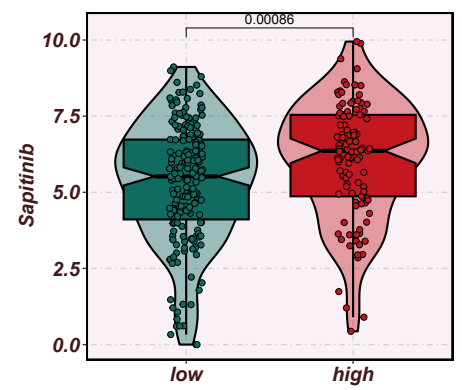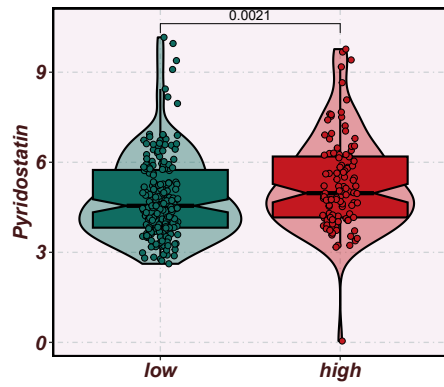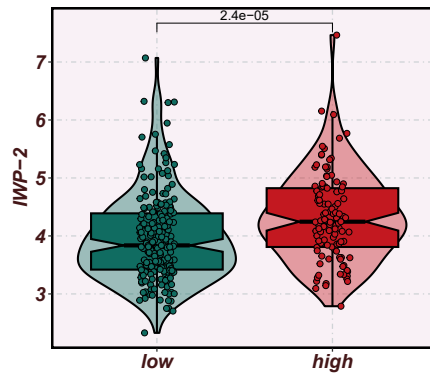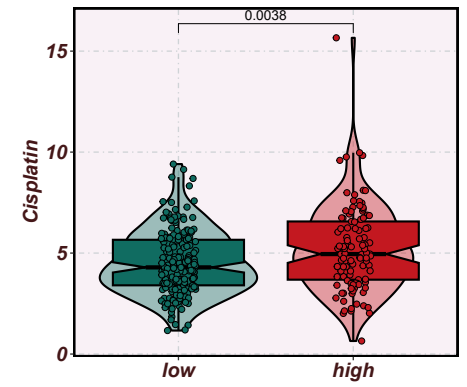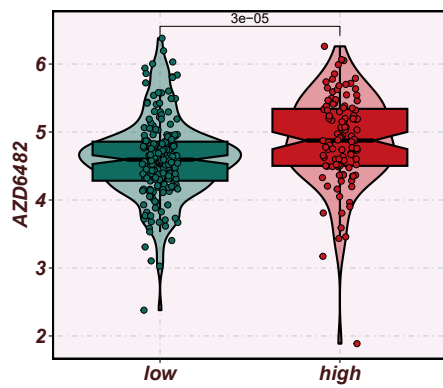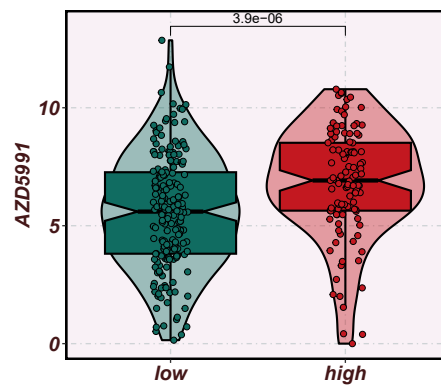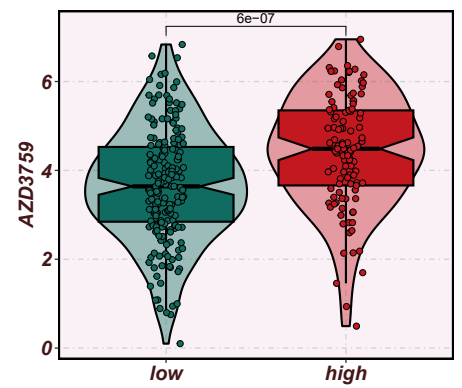

Supplement: Supplementary file 7 — Supporting Information 7 Figure S7: Box plots compare the sensitivity of the groups to nine chemotherapy drugs. [file HUMU-2026-8140041-s006.pdf]
