# Supplementary material for: Exploring the Dynamic Changes of Intercellular Connections in Cervical Cancer: Insights From Transcriptomic Data Combined With Single‐Cell Sequencing
Source: Hum Mutat. 2026 Jan 22;2026:8140041. doi: 10.1155/humu/8140041 (PMC12828070; doi:10.1155/humu/8140041)
Supplement: Supplementary file 8 — Supporting Information 8 Table S1: Sequences of qRT‐PCR primers or shRNA. [file HUMU-2026-8140041-s003.pdf]

Table S1 Sequences of qRT-PCR primers or shRNA

Gene	Species	Forward primer (5' -> 3')	Reverse primer (5' -> 3')
COL4A1	Human	GGACTACCTGGAACAAAAGGG	GCCAAGTATCTCACCTGGATCA
GAPDH	Human	ACAACTTTGGTATCGTGGAAGG	GCCATCACGCCACAGTTTC

Gene	Species	Sequences
shCOL4A1-1	Human	GCAGAGATGGTCTTGAAGGAT
shCOL4A1-2	Human	CCATGGATACTCTCTGCTCTA
shCOL4A1-3	Human	GCAATTACTACGCAAATGCTT
